# Telemedicine Chronic Viral Hepatitis C Treatment during the Lockdown Period in Romania: A Pilot Study

**DOI:** 10.3390/ijerph18073694

**Published:** 2021-04-01

**Authors:** Irina Paula Doica, Dan Nicolae Florescu, Carmen Nicoleta Oancea, Adina Turcu-Stiolica, Mihaela-Simona Subtirelu, Gindrovel Dumitra, Ion Rogoveanu, Dan Ionut Gheonea, Bogdan Silviu Ungureanu

**Affiliations:** 1 Gastroenterology Department, University of Medicine and Pharmacy of Craiova, 200349 Craiova, Romania; doicairinapaula@gmail.com (I.P.D.); dan.florescu@umfcv.ro (D.N.F.); ion.rogoveanu@umfcv.ro (I.R.); dan.gheonea@umfcv.ro (D.I.G.); bogdan.ungureanu@umfcv.ro (B.S.U.); 2 Pharmacy I Department, University of Medicine and Pharmacy of Craiova, 200349 Craiova, Romania; carmen.oancea@umfcv.ro; 3 Pharmacoeconomics Department, University of Medicine and Pharmacy of Craiova, 200349 Craiova, Romania; mihaela.subtirelu@umfcv.ro; 4 Family Medicine Department, University of Medicine and Pharmacy of Craiova, 200349 Craiova, Romania; dumitragino@yahoo.com

**Keywords:** telemedicine, lockdown, COVID-19, chronic hepatitis C, direct antiviral agents

## Abstract

The COVID-19 pandemic is currently delaying the process of chronic hepatitis C (HCV) eradication, since most of the chronic diseases are neglected. Thus, there is a need for alternative programs for HCV therapy implementation and disease monitoring. Our aim was to provide a multidisciplinary approach, so that HCV-infected patients from distant locations may benefit from HCV antivirals during the COVID-19 outbreak and within the lockdown period in Romania. Previously diagnosed HCV patients willing to participate in this telemedicine pilot study were included. Patient characteristics and medical adherence were assessed and compared to the year preceding the pandemic. We proposed a multidisciplinary approach by using a telemedicine program for HCV therapy monitoring. Patients also received a satisfaction questionnaire after delivering the sustained virologic response (SVR) result. A total of 41 patients agreed to participate in this study. The medication adherence was 100% for patients included in the telemedicine group, with a statistically significant difference from the medication adherence of the patients treated in 2019. The satisfaction item score was 4.92 out of 5 and our results (r = −0.94, *p* < 0.0001) suggested that older patients embraced the telemedicine program less, but with the same success in terms of SVR (100%) and medication adherence (100%). Our pilot study offers the first example of a telemedicine program in Romania for HCV therapeutic management. During the lockdown period, telemedicine has served as a reliable tool and novel alternative for conventional monitoring of patients treated with direct antiviral agents and should be further considered even following the pandemic.

## 1. Introduction

Chronic hepatitis C (HCV) affects over 71 million people worldwide; according to the World Health Organization (WHO), less than 5% are aware of their status [1]. The death toll due to viral hepatitis is still high, owing to its evolution to cirrhosis and its complications. Thus, proper screening methods and direct access to the new direct antiviral agents (DAA) are being developed to cover all the shortcomings of the HCV infection [2].

According to the WHO organization, nine countries are close to reaching the goal of HCV elimination by 2030 [3]. However, the success of this goal is at risk due to COVID-19, which has shifted medical focus towards limiting the pandemic’s effects. Since its spread in Europe, the objective of medical systems has been to avoid becoming overwhelmed by the large number of patients affected by COVID-19, which has shifted focus away from other diseases [4]. We observe a similar phenomenon in Romania, where the first case of COVID-19 was diagnosed on the 26th February 2020, which was followed by a general lockdown on March 16th, 2020 [5]. Thus, during this period the medical system focused exclusively on responding to major emergencies, and thus chronic disease has had to be managed using a different approach [6,7,8].

HCV patients in Romania are currently benefiting from two DAA-specific regimens provided by a national public health program, and only in cities with a university hospital [9]. This makes it difficult for patients from other regions to benefit from these therapeutic options.

Telemedicine has been embedded as an alternative in medicinal care by using store-and-forward information, remote monitoring and real-time long-distance interaction for patients that are not available for an on-site consultation [10]. Several studies have proven its effectiveness in patient–physician remote disease and treatment monitoring [11,12]. HCV infection therapy management was tested by telemedicine means in hard-to-reach locations as well as in correctional facilities, with positive results [13,14]. Thus, telemedicine concepts such as interactive telemedicine, store-and-forward set-up, as well as patient’s remote monitoring might be valid options during the pandemic to improve patient health status. However, challenges still prevail, since face-to-face interaction covers more information in the doctor–patient relationship.

There is a need for new approaches to the treatment of liver disease both during the pandemic and thereafter, so that viral hepatitis C progression may be limited. Facing the possible danger of COVID-19 and also seeing themselves not benefiting from the new DAAs, HCV patients might be affected in the long-term. Our aim was to provide a multidisciplinary approach, so that HCV-infected patients from distant locations may benefit from HCV antivirals. In this pilot study, we focused on implementing a telemedicine approach in HCV therapeutic management during the COVID-19 outbreak within the lockdown period in Romania.

## 2. Materials and Methods

### 2.1. Study Cohort and Design

**Figure 1:**
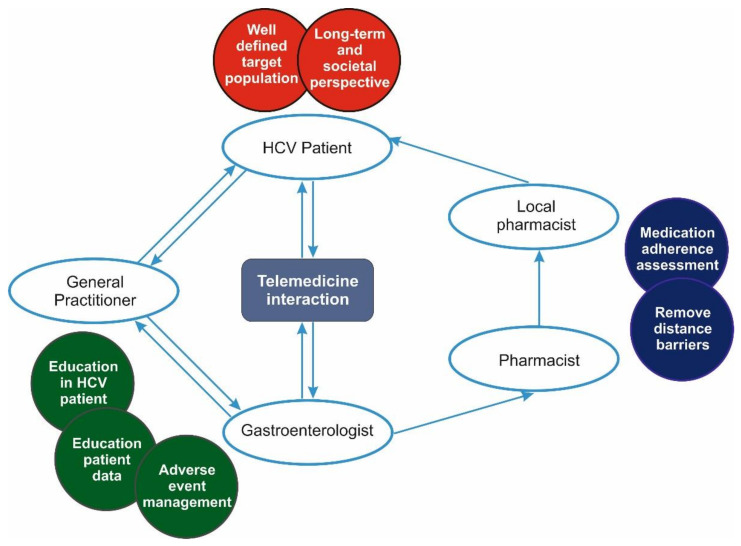
Study’s design.

The University County Hospital of Craiova Romania represents one of the eight delegated places to ensure DAA treatment for HCV-infected patients and covers a five-county region with an estimated population of 2,300,792 people. We previously implemented a monitoring platform for inflammatory bowel disease patients based on the store-and-forward concept [15], wherein a conceptual program for telemedicine was established using storage data, text messages, and freely available video applications. A multidisciplinary team was implemented, consisting of prescribing physicians, local general practitioners (GP) and pharmacists, as seen in Figure 1.

This study was conducted according to the guidelines in the Declaration of Helsinki, and approved by the Ethics Committee from The University of Medicine and Pharmacy of Craiova, Romania (Nr. 87/2020). All patients were informed and signed an “information form” as well as an “acceptance form”, according to national laws and the emergency state established during the COVID-19 pandemic [16,17,18].

### 2.2. Patients’ Characteristics

All patients included were previously diagnosed with HCV infection and were monitored by their GP. Patients received the blood work required for therapy inclusion criteria from their local GP, and according to the national guidelines for HCV therapy. The results and therapeutic decision were interpreted by gastroenterologists from Craiova County Hospital. The following parameters were assessed for DAA therapy inclusion.

The two available therapeutic options, dasabuvir with ombitasvir, paritaprevir, and ritonavir as well as sofosbuvir were prescribed according to possible drug interactions and patient compliance. Treatment varied according to the fibrosis stage, from 8 to 12 weeks. Three months after therapy, SVR blood tests were harvested, and since HCV genotype in Romania is reported to be genotype 1b, a high SVR is expected. Communication between pharmacists made it possible for medication to be transferred from one unit to another so that the patient could receive treatment.

We used anonymized individual patient data reported by community pharmacies from Dolj county at the Health Insurance House (HIH) of Dolj, and following the legal requirements for personal data protection. The patients’ data, extracted from the HIH of Dolj for 2019 and 2020, included the following demographic and treatment information: gender, age, health insurance type, date of prescription, international non-proprietary name (INN) and number of tablets for every dispensed prescription.

### 2.3. COVID-19 Period

We included patients that benefitted from DAA regimens during the lockdown period in Romania, which was between 15 March 2020 and 15 May 2020. We assessed therapeutic response, complication, and medication adherence, and compared it to 2019 HCV-treated patients for the same period of time through direct clinical meetings.

### 2.4. Telemedicine Protocol

Our approach comprised a multidisciplinary team (gastroenterologist, GP, and pharmacist) for remote interaction and monitoring of HCV patients so that they may obtain a sustained virologic response at the end of therapy. Distant patients who had been previously diagnosed with HCV were monitored and investigated by the local GP according to the gastroenterologist’s indication. Each patient forwarded the results to the physician (B.S.U., F.D.N.), who scheduled them for an initial remote consultation and treatment decision. Physician–patient interaction was comprised three sessions of video calls, which included an investigation assessment, DAA indication and side effects, as well as the patient’s adherence and compliance. Prescribing physicians contacted their pharmacists so that medication would be delivered to the local pharmacy, and every patient received a specific text message via mobile phone indicating when their medication was ready to collect. The study’s end-point was considered when physicians received the SVR blood tests and confirmed the results to the patient.

### 2.5. Telemedicine Satisfaction Questionnaire

The Telemedicine Satisfaction Questionnaire (TSQ) was used to assess satisfaction with telemedicine after Romanian translation and cultural adaptation had taken place [19]. The TSQ has 14 items rated on a 5-point Likert scale (“strongly disagree”, “disagree”, “neither agree nor disagree”, “agree”, “strongly agree”) with a good internal validity and consistency [20]. A maximum overall score of 5 can be obtained, indicating the maximum level of satisfaction with telemedicine. The other three maximum scores of 5 corresponded to quality of care provided, similarity to in-person / face-to-face interaction and perception of the interaction.

### 2.6. Medication Adherence

Treatment adherence was estimated as the proportion of the days covered (PDC), defined as the percentage of ∑ tablets dispensed/∑ tablets prescribed [21].

### 2.7. Statistical Analysis

We compared the patients’ characteristics and HCV treatment outcomes for two groups of patients: the group of patients with HCV treatment prescribed between March to May 2019, and the group of patients with HCV treatment prescribed during the lockdown phase (March–May 2020) who were included in the telemedicine study. We used Mann–Whitney test (for continuous variables) or Fisher’s exact test (for categorical variables). The Spearman correlation coefficient was calculated to assess the relationship between the level of telemedicine satisfaction and other patient characteristics. Given that the Cronbach’s alpha (α) value was considered to have several limitations when examining internal consistency or reliability [22], we reported the omega value (ω) [23]. We conducted statistical analysis using GraphPad Prism 9.0.0 software (GraphPad Software, LLC, San Diego, CA, USA) and the MBESS package in R software (R foundation, Vienna, Austria) [24].

## 3. Results

Several 223 HCV patients were treated between March–May 2019, and 41 entered the pilot study during the lockdown period in 2020. Table 1 shows no differences in gender and age between the two groups of patients. More employees were included in the lockdown phase than in the same period of 2019, and more pensioners in the 2019 period, as considered in the percentages of total observations in Table 1.

There was an increased delay in starting HCV treatment for new patients in 2020 than was observed in 2019: The first prescription of 2020 was issued on the 20th March, and the 3rd of January in 2019. This is the reason an increased number of new patients were included in the national HCV program during March 2020 than in March 2019 (as percentages of total patients, *p* = 0.037).

The medication adherence was 100% for patients included in the telemedicine group, with a statistically significant difference from the medication adherence of the patients treated in 2019.

To assess the results of our pilot study, we evaluated the sustained virological response and patient satisfaction with the telemedicine program. The McDonald’s omega value (ω = 0.866) confirmed that the TSQ internal consistency is good. Table 2 shows the average TSQ item score was 4.92 out of 5, with average scores of 4.84, 4.94, and 4.98 out of 5 in terms of quality of care provided, similarity to in-person/face-to-face interaction, and perception of the interaction, respectively. The results showed a negative correlation between TSQ score and age (*r* = −0.94, *p* < 0.0001), suggesting that older patients embraced the telemedicine program less, but with the same success in terms of sustained virological response (100%) and medication adherence (100%).

Most patients used mobile phones for connectivity (89%), and only 11% used other means of communication to access video, such as tablets or laptops. Across six meetings, we encountered internet connection problems and had to reschedule.

## 4. Discussion

In this section we report the first Romanian experience in using telehealth for HCV management. We reached our objective of treating HCV patients with DAA during the COVID-19 lockdown period in Romania via a combination of remote monitoring, medical reports transfer, as well as direct medication transfer within hard-to-reach regions. Telemedicine implementation is a challenge, as patients may be reluctant at first. We hypothesize that a major factor in our success was that patients had previously been diagnosed with an HCV infection and this was not part of a screening program, which may have proven more challenging.

Our pilot study suggests that telemedicine may be successfully implemented as a HCV treatment in Romania. As WHO suggested [25], by involving a multidisciplinary team and using the telemedicine concept, we overcame the disruption that COVID-19 pandemic had on HCV patients. With patients neglecting their disease, or considered clinically as nonemergent consultations, the COVID-19 pandemic developed a potentially negative impact on gastroenterologists [26], HCV elimination programs [27] and patients with chronic liver disease [28]. This study included only previously investigated HCV patients treated during the lockdown period in Romania, when travelling between cities had to be avoided. Thus, we promoted the use of telemedicine for HCV therapy management and assessment. Our results show that SVR was 100% for all included patients, regardless of the medication used. This may be related to several aspects. Firstly, most of the patients that joined our study were employees, which means they are still active people and more familiar with available technology. Moreover, the success of the previously treated patients, with few therapy side effects, made them more open to the treatment. Conversely, since the COVID-19 pandemic limited their access to travelling and direct communication, this study offered a reliable option to ensure treatment was followed. The design of our study involved both the GP and pharmacist, which helped the patient access local treatment. Scheduled 15 min video calls made by the hepatologist proved to be efficient in answering the patient’s questions regarding the treatment and following the patient’s status. Thus, the telemedicine program offered the same advantages as clinical visits.

HCV telemedicine programs were used in different countries over the world and proved that therapeutic success was similar, as if patients had been directed to a hepatology clinic [29]. Along with the pandemic, this concept was used for other diseases, especially in disease monitoring [30,31]. Thus, this may be a starting point for national programs and a more widespread implementation of the concept. However, general practitioners should be well trained and more familiar with this type of management, since they are the ones that frequently communicate with patients and suggest if a visit to a specialist is necessary. We proposed a protocol, which involved a pharmacist, since in Romania the HCV specific medication was only available in University Hospital cities, according to the national program. Pharmacist involvement has been considered for HCV treatment delivery as well as screening programs, with pharmacies being used at HCV antibody testing points [32]. Thus, they might also be integrated into a remote–distant program to help HCV elimination at the community level [33,34].

Ensuring multidisciplinary HCV management may address some flaws in disease management, especially throughout the pandemic. Direct communication between medical practitioners might aid patients in reaching SVR, since monitoring may be more accurate. While the role of the hepatologist is to prescribe the treatment, our scenario involved the local GP for local monitoring and community pharmacist to allow access to DAA therapy. Thus, inter-relations between local GPs, physicians, pharmacists help to seed a more trusting environment for HCV control management.

Patients’ experience with telemedicine was assessed to compare to face-to-face interaction, and the perception of interaction. In the era of modern communication, teleconsultation offered a major alternative for direct clinic visits in order to reduce the risk of COVID-19 infection in waiting rooms, as well as allaying the fear of direct medical interaction. Perhaps the most challenging aspects were to surpass the technical problems that may be encountered during a video consultation. The patients we included were not specifically trained for this type of interaction with a physician, and they were asked, therefore, to write down every question or any changes they might face during the treatment. Furthermore, most of the patients used their phones for interaction, and the internet connection was difficult to establish in just six live meetings, where patients were then rescheduled. The general satisfaction was high, especially in younger and active patients, even though 100% SVR and medical adherence were obtained.

Providing a telemedicine program changes the delivery health status and provides a strong relationship between clinician and patient, which is required [35,36]. This is the first reported study of telemedicine in treating HCV patients in Romania, and may be considered a starting point even outside the COVID-19 pandemic, due to the high response to available therapy. However, it is unclear if the patient’s satisfaction would be the same after the pandemic, and further studies should be embedded for a better implementation of programs. Physicians included in this study had to adapt to ensure HCV management, since their clinical duties were limited. Thus, alternative measures with lower exposure risk, enabled DAA adherence via telemedicine’s involvement. Treatment experience in previous years, along with available medical data provided by the GPs, helped physicians in the decision-making process.

Our study was limited by the small number of patients included. While all patients were HCV genotype 1b, SVR was expected to be high. However, this does not mean that the program might not be implemented for other genotypes. Only two DAA regimens were used according to the national guidelines and their availability. Patient–physician communication was done according to patient familiarity with the free video application on offer, since preparation and training was not possible due to the pandemic. Thus, this type of interaction limited physical examination. Chronic patients require a strong connection with the physician, and this might also be an important limitation to note. However, the COVID-19 pandemic accelerated the use of telemedicine, and since clinical visits were limited, patients were satisfied to communicate with the physician and receive DAA therapy (as shown by our results). For future implementation following the pandemic, improvements may be needed. Furthermore, most patients used only mobile phones for live sessions, and no consistent internet or broadband connection could be used in every situation. Furthermore, we did not perform a cost-analysis, though other studies have shown important savings by using telemedicine in HCV treatment [37]. While this was a pilot study, physician satisfaction with using telemedicine was not assessed. Barriers in adopting telemedicine in Romania for HCV management should be further studied so that more patients may benefit from DAA therapy, and so that physicians may be able to adapt their work.

## 5. Conclusions

During the lockdown period, telemedicine served as an important tool and potential alternative to the conventional monitoring of patients treated with DAA. Our setting, involving a multidisciplinary team for distantly located patients, helped to reach SVR. Prescribing physicians, local GPs and pharmacists played a pivotal role and proved to be a safe and effective approach to improve HCV management. Interdisciplinary collaboration may help disadvantaged communities by promoting telemedicine care and offer new pathways for disease monitoring and the achievement of therapeutic goals. Our pilot study promotes both a multidisciplinary approach and the future use of telemedicine in Romania for HCV treatment, laying a pathway for wider implementation at a national level.

## Figures and Tables

**Table 1:** Patient characteristics with comparison between pre-COVID phase and lockdown phase.

Characteristics	Pre-COVID Phase March–May 2019 (*n* = 223)	Lockdown Phase March–May 2020 (*n* = 41)	*p*-Value
Age (years)			0.9819
Mean ± SD	61.6 ± 11.4	62.1 ± 11.1	
Range	19–82	34–86	
Gender (n, %)			0.6904
Men	52, 23%	11, 27%	
Women	171, 77%	30, 73%	
Health insurance type (n, %)			0.0232 *^,a^
Employee	34, 15%	9, 22%	
Pensioner	146, 66%	19, 46%	
Co-insured	23, 10%	5, 12%	
Direct payment	6, 3%	3, 7%	
Social aid	5, 2%	3, 7%	
Other	9, 4%	2, 5%	
Medication adherence (Mean ± SD)	94.7 ± 15.4	100 ± 0	0.0328 *
New patients/month (n, %)			0.037 *^,b^
March	87, 39%	22, 54%	
April	77, 35%	9, 22%	
May	59, 26%	10, 27%	

^a^, pre-COVID phase pensioner vs. lockdown phase pensioner; ^b^, pre-COVID phase March vs. lockdown phase March. *, significantly different if *p* < 0.05.

**Table 2:** Outcomes for telemedicine patients.

Outcomes	Lockdown Phase–Telemedicine Patients (*n* = 41)
Treatment (n, %)	
ombitasvir/paritaprevir/ritonavir + dasabuvir	7, 17%
Ledipasvir + sofosbuvir	34, 83%
Sustained virological response (n, %)	41, 100%
Telemedicine satisfaction (mean ± SD)	
General satisfaction	4.92 ± 0.11
Quality of care provided	4.84 ± 0.17
Similarity to in-person face-to-face interaction	4.94 ± 0.10
Perception of the interaction	4.98 ± 0.16

## Data Availability

The authors declare that the data in this research is available.
